# Authenticity as a Resilience Factor Against CV-19 Threat Among Those With Chronic Pain and Posttraumatic Stress Disorder

**DOI:** 10.3389/fpsyg.2021.643869

**Published:** 2021-04-28

**Authors:** David E. Reed, Elizabeth Lehinger, Briana Cobos, Kenneth E. Vail, Paul S. Nabity, Peter J. Helm, Madhwa S. Galgali, Donald D. McGeary

**Affiliations:** ^1^Center of Innovation for Veteran-Centered and Value-Driven Care, VA Puget Sound Health Care, Seattle, WA, United States; ^2^Department of Psychiatry and Behavioral Sciences, Texas Health Science Center at San Antonio, San Antonio, TX, United States; ^3^Department of Psychiatry and Behavioral Sciences, University of Washington, Seattle, WA, United States; ^4^Department of Psychology, University of Texas at San Antonio, San Antonio, TX, United States; ^5^Department of Psychology, Cleveland State University, Cleveland, OH, United States; ^6^Department of Psychological Sciences, University of Missouri, Columbia, MO, United States; ^7^Department of Rehabilitation Medicine, University of Texas Health Science Center at San Antonio, San Antonio, TX, United States

**Keywords:** COVID-19 pandemic, CV-19 threat, CV-19 impact, authenticity, pain-related disability, PTSD

## Abstract

**Objective:**

The novel coronavirus (2019; CV-19) is linked to increases in emotional distress and may be particularly problematic for those with pre-existing mental and physical conditions, such as chronic pain and posttraumatic stress disorder (PTSD). However, little empirical research has been published on resilience factors in these individuals. The present study aims to examine authenticity as a resilience factor among those with chronic pain and/or PTSD.

**Methods:**

Prior to the national response to the pandemic (January 10-24, 2020), participants were screened for pain-related disability (Oswestry Disability Index; ODI) and PTSD symptoms (Posttraumatic Checklist for DSM-5; PCL-5), and on the basis of those responses were categorized into one of four groups: healthy, chronic pain only, PTSD only, or comorbid chronic pain and PTSD. During the CV-19 pandemic (May 5-May 13, 2020), participants responded again to the ODI and PCL-5, in addition to the Wood Authenticity Scale, Brief Pain Inventory, and items related to the CV-19 pandemic.

**Results:**

A total of 110 participants (54.55% women), aged 42.19 (*SD* = 13.16), completed the survey during the pandemic. The comorbid group endorsed higher levels of CV-19 Threat and Impact compared to all other groups. Authenticity moderated this relationship relevant to CV-19 Threat among those in the chronic pain only group, and not in any other group.

**Conclusion:**

The comorbid group endorsed higher levels of CV-19 Threat and Impact compared to all other groups. Importantly, greater authenticity was associated with less CV-19 Threat in the chronic pain only group, and not in any other group. The present study also highlights the importance of engaging authentically for those with chronic pain during the pandemic.

## Introduction

The novel coronavirus (2019; CV-19) has claimed the lives of over 550,000 individuals in the United States (World Health Organization [WHO], 2020). In addition, increases in suicidality, anxiety, and depression have been documented during the pandemic (Czeisler et al., 2020), highlighting the pandemic’s psychological toll. Individuals with physical or psychological pre-existing conditions, such as chronic pain (defined as experiencing pain for at least 3 months) and/or posttraumatic stress disorder (PTSD), may be significantly impacted by the pandemic (Merskey and Bogduk, 1994; Treede et al., 2015; Pfefferbaum and North, 2020). Nevertheless, little empirical research has been published specifically on these populations during the pandemic. Moreover, there is a paucity of research on understanding how resilience factors may help patients with chronic pain and PTSD navigate through the effects of the pandemic. Resilience is adapting to novel stressors in a way that prevents long-term mental health issues (Kalisch et al., 2017). It is a learned set of behaviors, not an innate factor that cannot be changed (Kalisch et al., 2017). Capturing data on putative resilience factors in the midst of adversity, such as the current CV-19 pandemic, is critical to our understanding of real-world resilience in these populations. The present work aims to fill this gap by assessing authenticity as a resilience factor among those with chronic pain only, PTSD only, and comorbid chronic pain and PTSD.

Among the general population, research has focused on CV-19 pandemic-specific anxieties, in addition to CV-19 pandemic-specific financial and emotional burdens (Conway et al., 2020; Guo et al., 2020; Liu et al., 2020), defined by Conway et al. (2020) as CV-19 Threat and CV-19 Impact, respectively. The financial impact of the CV-19 pandemic among the general population is linked to higher symptoms of PTSD and depression (Guo et al., 2020), and anxiety specific to CV-19 has been linked to depression, anxiety, alcohol use, and PTSD (Liu et al., 2020; Rodriguez et al., 2020).

Individuals with mental and physical health conditions are constantly managing the stress of their disorders. The physiological and psychological burden, or “allostatic load,” may result in difficulties processing and managing additional stressors (McEwen, 1998; Sterling and Eyer, 1988). For example, pain-related disability worsens when depression or PTSD is also included in the clinical picture along with pain (Benedict et al., 2020; McGeary et al., 2020), and cardiovascular and metabolic issues are linked to PTSD (Ryder et al., 2018). The pandemic serves as an additional stressor for those with chronic pain and/or PTSD. Therefore, individuals with more severe mental and physical health symptoms may endorse more severe perceptions of CV-19 anxiety and CV-19 impact. However, because of authenticity’s positive effects on mood (Grijak, 2017), feeling authentic may promote resilience and help ameliorate these effects.

Resilience is an adaptive and dynamic process, whereby stressors result in cognitive, affective, and behavior changes that promote mental health (Kalisch et al., 2017, 2019). In a way, resilience is about either developing and enacting one’s new authentic self, or ensuring that one’s authentic self is not affected negatively. Authenticity is defined as congruence between one’s internal physiological states (e.g., feelings of happiness), accurate awareness of these states, and engaging in behaviors that are appropriate to those internal states and corresponding awareness (Wood et al., 2008). Inauthenticity may be felt when internal psychological states do not match with one’s awareness of these states (i.e., self-alienation) and/or when awareness of these states are not congruent with behaviors (i.e., inauthentic living; Wood et al., 2008). As such, authenticity entails emotional states, cognitions, and behaviors. Higher levels of authenticity have been linked to increased psychological well-being, decreased anxiety, and decreased depression (Grijak, 2017).

In a longitudinal study examining authenticity, life satisfaction, and distress across two time points, Boyraz et al. (2014) found that authenticity predicted greater life satisfaction and less distress six weeks later. Moreover, the relationships between authenticity, life satisfaction, and distress were all significant at Time 2, suggesting that authenticity served as a concurrent and future resilience factor. Results are congruent with cross-sectional studies showing that authenticity is linked to lower levels of anxiety, depression, and loss of control, and greater meaning in life and psychological well-being (Vess et al., 2016; Grijak, 2017).

Theorists posit that one’s mortality is at the core of the distress related to the CV-19 pandemic (Pyszczynski et al., 2020). Although viewing one’s future as limited may promote negative affect (Grühn et al., 2016), it is also an opportunity to harness positive outcomes (Vail et al., 2012; Reed, 2020). Authenticity may buffer against negative affect associated with limited time perspectives in the future during the pandemic. Indeed, Davis and Hicks (2013) published a series of experimental studies showing how authenticity helps promote hope when experiencing limited time perspectives. In addition, Bryan et al. (2017) found that authenticity attenuated the relationship between loneliness and depression, anxiety, and physical symptoms. A recent longitudinal study provided compelling evidence for authenticity as a buffer against collective trauma within the context of Hurricane Harvey. The events of Hurricane Harvey, similar to the CV-19 pandemic, affected a large group of individuals and significantly altered their way of living. Among a sample of individuals of which many had experienced Hurricane Harvey, Maffly-Kipp et al. (2020) showed that inauthenticity at an earlier time point contributed to higher stress at a later time point. These results are congruent with the longitudinal analyses of Boyraz et al. (2014) and showed that stressors that alter daily existence are attenuated by perceived authenticity. Authenticity as a resilience factor may be particularly crucial for individuals who are forced to alter their preferred way of being due to mental or physical health conditions, such as those with chronic pain and/or PTSD.

Individuals diagnosed with chronic pain and/or PTSD potentially experience a disruption of their sense of self, often precipitated by the loss of social roles (e.g., employment) and an inability to engage in life as they desire (Morley, 2008; Mitchell et al., 2020; Packham et al., 2020). Avoidance, social isolation, and/or pain-related disability may prevent individuals from engaging in behaviors that provide a sense of authenticity (i.e., engaging in life consistent with how one views themselves; Wood et al., 2008). This is particularly problematic because lower levels of authenticity are associated with more distress, anxiety, and depression, and lower levels of well-being (Sheldon et al., 1997; Grijak, 2017).

Chronic pain does not merely interfere with one’s life; individuals with chronic pain experience a disruption of identity and self-definition (Morley, 2008). Indeed, chronic pain may be considered an external influence that potentially disrupts one’s perceived authenticity, as described by Wood et al. (2008). Adults with chronic pain have difficulty recognizing their emotions, which is associated with higher pain severity, pain disability, and distress (Aaron et al., 2019; Blaettler et al., 2019). In turn, this may result in individuals with chronic pain acting in ways that are not congruent with how they view themselves (e.g., not engaging with their grandchildren due to pain; Smith and Osborn, 2007), thus contributing to inauthenticity. Moreover, individuals may experience their emotions and thoughts associated with chronic pain as inauthentic. In all, chronic pain acts as a problematic external influence that forces individuals to engage in life in a different way relative to their premorbid and/or preferred emotional, physical, and social functioning (McWilliams et al., 2003; Morley, 2008; De Heer et al., 2014; Packham et al., 2020).

Similarly, PTSD is defined by avoidance of internal and external trauma reminders, negative alterations in cognitions and mood, hypervigilance, and intrusive symptoms (American Psychiatric Association [APA], 2013). Avoiding previously valued behaviors (e.g., going to play in a park or going out to eat) in the service of preventing thoughts and emotions related to a traumatic experience may be felt as an inauthentic behavior. In addition, loss of trust and security, both part of the PTSD sequelae (Bell et al., 2019; Ehlers and Clark, 2000), may prevent individuals from engaging with others in a way that feels natural and authentic.

Individuals with comorbid chronic pain and PTSD are particularly susceptible to disruptions of identity (Reed et al., 2021). Not only are they managing the symptoms of a physical and mental health disorder simultaneously, but symptoms of pain and PTSD may maintain each other (mutual maintenance model; Sharp and Harvey, 2001). For instance, Feinberg et al. (2017) found that hyperarousal and intrusion symptoms at 6 weeks after an emergency department (ED) visit mediated the relationship between pain symptoms during the ED visit and pain symptoms 1 year after the ED visit. Moreover, individuals with chronic pain and PTSD endorse higher levels of pain severity, depression, and healthcare utilization compared to those with pain but without PTSD (Benedict et al., 2020). In line with the theory of allostatic load, it is likely that individuals with comorbid chronic pain and PTSD would experience the pandemic differently than those with one or none of these disorders, endorsing higher levels of CV-19 Threat and Impact. Living authentically may ameliorate CV-19 Threat and Impact among this population, however.

If engaging in life authentically is associated with positive outcomes (Grijak, 2017), then individuals with higher levels of authenticity would presumably be protected from anxieties and burdens associated with the pandemic. Therefore, we hypothesized that authenticity would be associated with lower levels of CV-19 Threat and Impact. The addition of chronic pain, PTSD, or comorbid chronic pain and PTSD increases one’s allostatic load and vulnerability to an additional stressor, such as the pandemic. Therefore, we hypothesized that the relationship between authenticity and CV-19 Threat and Impact would be weaker (i.e., an interaction between group and authenticity) for individuals in the chronic pain only and PTSD only groups (compared to the healthy group) and in the comorbid chronic pain and PTSD only group (compared to all other groups).

## Method

### Procedure

The present research utilized data collected during the CV-19 pandemic as part of a larger longitudinal quasi-experimental study. Data were collected using CloudResearch (Litman et al., 2017), a web interface designed to more easily recruit reliable and valid online data from several clinical populations. For overall study design^1^. Prior to the national response to the pandemic (January 10-24, 2020), participants were screened for pain-related disability (Oswestry Disability Index; ODI) and PTSD symptoms (Posttraumatic Checklist for DSM-5; PCL-5), and on the basis of those responses were categorized into one of four groups. The healthy group was comprised of individuals in the lower quartile on the ODI (8% pain disability or less) and PCL-5 (15 or less). The chronic pain only group endorsed experiencing pain for at least 3 months and at least 21% pain disability (which is indicative of moderate disability associated with everyday movements; Michigan State University, 2020) and in the lower quartile on the PCL-5. Individuals in the PTSD only group were in the lower quartile of pain disability and endorsed 37 or higher on the PCL-5. Research suggests 37 is an optimal cut-off score when determining a PTSD diagnosis when over-diagnosing is a concern (i.e., high specificity; Blevins et al., 2015). Individuals in the comorbid chronic pain only and PTSD only group endorsed moderate to severe pain disability and at least 37 on the PCL-5. If individuals did not fall into these categories, they were excluded from participation. For consort chart^2^. For the present study, only scores collected at Time 2 (during the CV-19 pandemic) were used. For more detailed information on the specifics of the present study design, including pre-registered hypotheses^3^. The present study was completed with approval from the University of Texas Health Science Center IRB.

### Participants

A total of 110 participants (60 women; 54.55%) completed assessments during the CV-19 pandemic between May 5 and May 13, 2020. On average, participants were 42.19 (*SD* = 13.16) years old, with an age range of 22 to 82. There were statistically significant differences in age across groups, *F*(3,106) = 7.73, *p* < 0.001, with Tukey’s HSD bias correction indicating the chronic pain group (*M* = 50.18; *SD* = 13.39) was older than the healthy group (*M* = 37.66; *SD* = 12.99; *difference* = 12.52 [*95% CI:* 4.75, 20.29], *p* < 0.001), PTSD group (*M* = 37.18; *SD* = 10.61; *difference* = –12.99 [*95% CI:* –21.63, –4.36], *p* = 0.001), and comorbid group (*M* = 41.45; *SD* = 9.70; *difference* = –8.72 [*95% CI:* –17.36, –0.09], *p* = 0.047). Participants were predominantly White (*n* = 94; 85.45%) and either had a 4-year college degree (*n* = 34; 30.91%) or some college (*n* = 29; 26.36%) or. See Table 1 for all sample characteristics.

**TABLE 1:** Sample characteristics.

	**Healthy**	**Chronic Pain**	**PTSD**	**Comorbid**	***p***	**SMD**
	**Mean or Freq. SD or Percentage**	**Mean or Freq. SD or Percentage**	**Mean or Freq. SD or Percentage**	**Mean or Freq. SD or Percentage**		
*n*	32	34	22	22		
Age	37.66 (12.99)	50.18 (13.39)	37.18 (10.61)	41.45 (9.70)	<0.001	0.594
Race					0.048	0.646
African American	3 (9.4)	0 (0.0)	1 (4.5)	2 (9.1)		
Asian	6 (18.8)	0 (0.0)	0 (0.0)	2 (9.1)		
Decline to Answer	0 (0.0)	0 (0.0)	0 (0.0)	1 (4.5)		
Hispanic	0 (0.0)	1 (2.9)	0 (0.0)	0 (0.0)		
White/Caucasian	23 (71.9)	33 (97.1)	21 (95.5)	17 (77.3)		
Gender					0.570	0.220
Decline to Answer	0 (0.0)	0 (0.0)	0 (0.0)	1 (4.5)		
Female	18 (56.2)	17 (50.0)	12 (54.5)	13 (59.1)		
Male	14 (43.8)	17 (50.0)	10 (45.5)	8 (36.4)		
Sexual Orientation					0.148	0.449
Bisexual	2 (6.2)	2 (5.9)	5 (22.7)	2 (9.1)		
Gay or Lesbian	1 (3.1)	0 (0.0)	2 (9.1)	2 (9.1)		
Heterosexual	29 (90.6)	32 (94.1)	15 (68.2)	18 (81.8)		
Education					0.056	0.917
2-year College Degree	2 (6.2)	8 (23.5)	4 (18.2)	6 (27.3)		
4-year College Degree	17 (53.1)	8 (23.5)	5 (22.7)	4 (18.2)		
Decline to Answer	0 (0.0)	0 (0.0)	0 (0.0)	1 (4.5)		
Doctoral Degree	1 (3.1)	0 (0.0)	0 (0.0)	1 (4.5)		
High School/GED	2 (6.2)	4 (11.8)	4 (18.2)	0 (0.0)		
Less than High School	1 (3.1)	0 (0.0)	0 (0.0)	0 (0.0)		
Master’s Degree	5 (15.6)	1 (2.9)	3 (13.6)	4 (18.2)		
Some College	4 (12.5)	13 (38.2)	6 (27.3)	6 (27.3)		

*SMD = standardized mean difference.*

### Measures

#### Demographics

Participants provided information related to gender, age, race, and education.

#### CV-19 Threat

Participants completed a 6-item questionnaire measuring how threatened or worried they were about CV-19 on a 7-point scale (1 = “Not True of Me At All” to 7 = “Very True of Me”). Examples of questions include “Thinking about the coronavirus (COVID-19) makes me feel threatened” and “I am afraid of the coronavirus” (Conway et al., 2020). Internal consistency of CV-19 Threat for the present study was α = 0.90.

#### CV-19 Impact

Participants completed a 9-item assessment measuring how participants were financially and psychologically impacted by the coronavirus on a 7-point scale (1 = “Not True of Me At All” to 7 = “Very True of Me”). Questions included: “The Coronavirus (COVID-19) has impacted me negatively from a financial point of view” and “I have lost job-related income due to the Coronavirus.” Items were summed for data analysis (Conway et al., 2020). Internal consistency of CV-19 Impact for the present study was α = 0.85.

#### Authenticity

The Wood Authenticity Scale is a 12-item assessment that measures three components of authenticity: authentic living, accepting external influence, and self-alienation (Wood et al., 2008). An example item is, “I am true to myself in most situations,” and participants responded on a 7-point scale (1 = “Does not describe me at all” to 7 = “Describes me very well”). Items were summed for a total score, and the internal consistency for the present study was α = 0.91.

#### Pain Disability

A modified version of the Oswestry Disability Index (ODI) measured an individual’s perception of disability due to pain using ten different daily life domains, such as lifting, walking, traveling, sitting, standing, sleeping, social life, and employment (Fairbank et al., 1980; Fritz and Irrgang, 2001). Participants completed the 10-item questionnaire on a 0-5 scale. A final percentage score was taken for each time point by taking the average of each item and multiplying that average by 100. Internal consistency of the ODI for the present study was α = 0.93.

#### PTSD

The Posttraumatic Stress Disorder Checklist for DSM-5 (PCL-5) was used to measure PTSD symptoms using the current Diagnostic and Statistical Manual’s criteria (Weathers et al., 2013). Individuals were asked to indicate a traumatic event (i.e., Criterion A event) in their lifetime and the extent to which they have been bothered by PTSD symptoms in the past month using a 20-item rating scale from a 0 to 4 scale (0 = “Not at all” to 4 = “Extremely”). Items were summed for data analysis. Internal consistency of the PCL-5 for the present study was α = 0.96.

#### Pain Severity

The Modified Brief Pain Inventory Short Form (mBPI-SF; Cleeland, 1991) measured the level of the participant’s pain severity by averaging 4-items of pain at its worst, least, average, and current pain (i.e., now; 0-10 scale). An example item is, “Please rate your pain by marking the number that best describes your pain at its worst in the last 24 hours.” Items were averaged for data analysis. Internal consistency for the mBPI-SF for the present study was α = 0.94.

### Data Analytic Strategy

In order to address study hypotheses, general linear models (GLM) were examined, one for each outcome (CV-19 Threat and CV-19 Impact), wherein Gaussian distributions were specified. Each model included Group and Authenticity as predictors, in addition to a Group by Authenticity interaction. Visual inspection of data plots indicated that a quadratic relationship may emerge between authenticity and the outcome of interest. Therefore, a quadratic authenticity term was entered into each model to account for curvilinear relationships. Authenticity was grand-mean centered in each model, and the centered variable was used to create the interaction and quadratic terms. Age (grand-mean centered) was included in the model as a covariate due to between-group differences. Regression estimates of GLMs were examined for significance. Correlations between variables of interest are also presented.

## Results

### Descriptive Statistics

Participants in the healthy group (*n* = 32) endorsed low levels of pain severity (*M* = 0.84; *SD* = 1.37), pain disability (*M* = 2.56%; *SD* = 4.38%), and PTSD symptoms (*M* = 4.19; *SD* = 5.86). Individuals in the chronic pain only group (*n* = 34) endorsed pain severity levels (*M* = 3.65; *SD* = 2.01) most consistent with mild pain (Serlin et al., 1995; Jones et al., 2007), and pain disability levels (*M* = 29.24%; *SD* = 18.08%) consistent with individuals with chronic pain. The chronic pain only group endorsed PCL-5 scores (*M* = 8.53; *SD* = 10.16) consistent with individuals without PTSD. Individuals in the PTSD only group endorsed an average pain severity rating of 2.22 (*SD* = 2.06) and pain disability of 9.36% (*SD* = 10.00%), consistent with individuals without chronic pain. The PTSD only group endorsed PCL-5 scores slightly below average of what would be consistent with a PTSD diagnosis (*M* = 30.09; *SD* = 17.20) but is close to a suggested cut-off score of 31-33 (Blevins et al., 2015). Pre-screening prior to the pandemic indicated PCL-5 scores were consistent with a PTSD diagnosis for those who completed both time points (*M* = 47.77; *SD* = 9.60). In addition, recommendations leave room for lowering the cut-off scores depending on the purpose of the assessment (U.S. Department of Veterans Affairs, 2020). Finally, participants in the comorbid group endorsed an average pain severity rating of 3.59 (*SD* = 2.27), pain disability of 33.18% (*SD* = 19.29%), and PCL-5 score of 38.23 (*SD* = 17.65), all of which are consistent with individuals who have comorbid chronic pain and PTSD. Average authenticity score was 68.78 (*SD* = 13.27) for the healthy group, 71.68 (*SD* = 9.90) for the chronic pain only group, 66.59 (*SD* = 15.55) for the PTSD only group, and 58.36 (*SD* = 16.59) for the comorbid group. Analysis of variance indicated significant group differences in authenticity (using Tukey’s HSD correction; *F*[3, 106] = 4.50, *p* = 0.005), with the comorbid condition endorsing less authenticity than the healthy group (*difference* = –10.42 [*95% CI:* –20.24, –0.60], *p* = 0.033) and chronic pain only group (*difference* = –13.31 [*95% CI:* –23.01, –3.61], *p* = 0.003).

### Outcomes

#### CV-19 Threat

A main effect of group emerged with CV-19 Threat as the outcome, *F*(3,100) = 2.79, *p* = 0.044, *partial* *η^2^* = 0.05. Regression analyses indicated that individuals in the comorbid group endorsed more CV-19 Threat compared to individuals in the healthy group (*b* = 6.98, *S.E.* = 2.78, *p* = 0.014, *95% CI:* 1.52, 12.43, *β* = 0.29). The chronic pain only group (*b* = 1.00, *S.E.* = 2.61, *p* = 0.703, *95% CI:*–4.12, 6.12, *β* = 0.05) and PTSD only group (*b* = –0.99, *S.E.* = 2.54, *p* = 0.697, *95% CI:*–5.96, 3.98, *β* = –0.04) did not differ in CV-19 Threat compared to the healthy group. Estimated marginal means are shown in Table 2. When Tukey’s HSD bias correction is applied for CV-19 Threat between-group differences, the differences between the healthy group and comorbid group are marginally significant (*p* = 0.059); differences between the PTSD group and comorbid group are significant (*p* = 0.041), and differences between the chronic pain group and comorbid group are non-significant (*p* = 0.162). There was no main linear effect of authenticity, *F*(1,100) = 0.29, *p* = 0.592, *partial* *η^2^* = –0.01, which did not support our hypothesis. Moreover, the quadratic term of authenticity was non-significant, *F*(1,100) = 0.61, *p* = 0.437, *partial* *η*^2^ = –0.00, as was the main effect of age, *F*(1,100) = 0.17, *p* = 0.684, *partial* *η*^2^ = –0.01.

**TABLE 2:** Estimated marginal means and Tukey’s HSD comparisons.

**Condition**	**CV-19 Threat Mean [95% CI]**	**CV-19 Impact Mean [95% CI]**
Healthy	27.97 [24.71, 31.23]^#^	30.69 [26.37, 35.00]
Chronic Pain	28.97 [25.23, 32.71]	30.41 [25.46, 35.35]
PTSD	26.98 [23.10, 30.86]*	32.34 [27.21, 37.47]
Comorbid	34.95 [30.53, 39.37]^#^*	43.83 [37.99, 49.67]^**^

*Indicates significant between-group differences of CV-19 Threat at *p* < 0.05; ^#^indicates marginally significant between-group differences of CV-19 Threat at *p* < 0.10; ^**^Comorbid condition endorsed significantly greater CV-19 Impact than all other groups at *p* < 0.05; there were no other between-group differences for CV-19 Impact.

We also hypothesized that the relationship between authenticity and CV-19 Threat would be dependent on group, with the healthy group showing the strongest relationship, followed by the chronic pain only and PTSD only groups, and then the comorbid group. Although we did not find this specific pattern, we did find that the authenticity and CV-19 Threat relationship was dependent on group. A marginally significant group by authenticity interaction emerged, *F*(3,100) = 2.50, *p* = 0.064, *partial* *η^2^* = 0.04. Regression analyses showed that the relationship between authenticity and CV-19 Threat significantly differed between the healthy group and chronic pain only group, *b* = –0.50, *S.E.* = 0.21, *p* = 0.019, *95% CI: –*0.91, –0.09, *β* = –0.30. A simple slopes analysis showed that for those in the chronic pain only group, as authenticity increased, CV-19 Threat decreased, *b* = –0.57, *S.E.* = 0.16, *p* = 0.001. This same relationship was not seen in the other groups (*p*s > 0.05). The overall model was significant, *F*(9,100) = 2.88, *p* = 0.005, with an adjusted *R*^2^ = 0.13. See Table 3 for regression estimates.

**TABLE 3:** Regression estimates – CV-19 Threat.

**Predictor**	**Estimate**	**Est. 95% CI**	**Std. Error**	***t*-statistic**	***p***
Intercept	28.65	[25.01, 32.29]	1.86	15.44	<0.001*
**Condition**					
Chronic Pain	1.00	[–4.12, 6.12]	2.61	0.38	0.703
PTSD	–0.99	[–5.96, 3.98]	2.54	–0.39	0.697
Comorbid	6.98	[1.52, 12.43]	2.78	2.51	0.014*
Authenticity (Linear)	–0.07	[-0.32, 0.18]	0.13	–0.54	0.592
Authenticity (Quadratic)	0.00	[–0.01, 0.01]	0.00	–0.78	0.437
Age	0.03	[–0.12, 0.18]	0.08	0.41	0.684
**Interactions**					
Authenticity*Chronic Pain	–0.50	[–0.91, –0.09]	0.21	–2.39	0.019*
Authenticity*PTSD	–0.02	[–0.38, 0.34]	0.18	–0.12	0.909
Authenticity*Comorbid	0.13	[–0.27, 0.53]	0.20	0.64	0.523

Healthy condition modeled as reference group; *p < 0.05.

#### CV-19 Impact

With CV-19 Impact as the outcome of interest, a significant main effect of group emerged, *F*(3,100) = 5.37, *p* = 0.002, *partial* *η^2^* = 0.11. Regression analyses showed that individuals in the comorbid group, compared to individuals in the healthy group, endorsed more CV-19 Impact (*b* = 13.14, *S.E.* = 3.68, *p* = 0.001, *95% CI:* 5.92, 20.36, *β* = 0.40). The chronic pain only group (*b* = –0.28, *S.E.* = 3.45, *p* = 0.935, *95% CI:* –7.05, 6.49, *β* = –0.01) and PTSD only group (*b* = 1.65, *S.E.* = 3.35, *p* = 0.623, *95% CI:* –4.92, 8.22, *β* = 0.05) did not differ from the healthy group. Estimated marginal means showed the same significance pattern when using Tukey’s HSD bias correction (see Table 2). Our hypothesis was not supported, as there was no main effect of linear authenticity, *F*(1,100) = 2.44, *p* = 0.122, *partial* *η^2^* = 0.01. Quadratic authenticity, *F*(1,100) = 1.29, *p* = 0.260, *partial* *η*^2^ = 0.00 and age were non-significant, *F*(1,100) = 0.00, *p* = 0.996, *partial* *η*^2^ = –0.01. The group by authenticity interaction was also non-significant, *F*(3,100) = 0.58, *p* = 0.626, *partial* *η*^2^ = –0.01, which also did not support our hypothesis of authenticity being differentially related to CV-19 Impact relevant to group. The overall model was significant, *F*(9,100) = 3.56, *p* = 0.001, with an adjusted *R*^2^ = 0.17. See Table 4 for regression estimates.

**TABLE 4:** Regression estimates – CV-19 Impact.

**Predictor**	**Estimate**	**Est. 95% CI**	**Std. Error**	***t*-statistic**	***p***
Intercept	31.97	[23.55, 40.40]	4.30	7.44	<0.001*
**Condition**					
Chronic Pain	–0.28	[–7.05, 6.49]	3.45	–0.08	0.935
PTSD	1.65	[–4.92, 8.22]	3.35	0.49	0.623
Comorbid	13.14	[5.92, 20.36]	3.68	3.57	0.001*
Authenticity (Linear)	–0.26	[–0.59, 0.07]	0.17	–1.56	0.122
Authenticity (Quadratic)	–0.01	[–0.02, 0.00]	0.01	–1.13	0.260
Age	0.00	[–0.19, 0.20]	0.10	0.01	0.996
**Interactions**					
Authenticity*Chronic Pain	–0.02	[–0.56, 0.52]	0.28	–0.06	0.954
Authenticity*PTSD	0.27	[–0.21, 0.75]	0.24	1.10	0.274
Authenticity*Comorbid	0.03	[–0.50, 0.55]	0.27	0.10	0.925

Healthy condition modeled as reference group; *p < 0.05.

#### Correlation Analyses

Correlation analyses collapsing across participant groups revealed the general relationships between variables included in the present study. Higher levels of authenticity were significantly related to less CV-19 Impact, *r*(108) = –0.25, *p* = 0.010, and less severe PTSD symptoms, *r*(108) = –0.49, *p* < 0.001. Higher levels of authenticity were marginally significantly related to lower levels of CV-19 Threat, *r*(108) = –0.18, *p* = 0.061, and pain-related disability, *r*(108) = –0.18, *p* = 0.058, and older age, *r*(108) = 0.17, *p* = 0.079. Higher levels of CV-19 Threat were significantly associated with higher levels of CV-19 Impact, *r*(108) = 0.49, *p* < 0.001, and pain severity *r*(108) = 0.21, *p* = 0.025, and marginally significantly related to more severe PTSD symptoms, *r*(108) = 0.17, *p* = 0.081. Being impacted by CV-19 was significantly associated with more pain-related disability, *r*(108) = 0.21, *p* = 0.028, PTSD symptoms, *r*(108) = 0.37, *p* < 0.001, and pain severity, *r*(108) = 0.26, *p* = 0.005. See Table 5 for all correlations.

**TABLE 5:** Correlations between variables of interest.

**Variable**	**1**	**2**	**3**	**4**	**5**	**6**	**7**
1. CV-19 Threat	-						
2. CV-19 Impact	0.49*	-					
3. Authenticity	–0.18^#^	–0.25*	-				
4. Pain-Related Disability	0.14	0.21*	–0.18^#^	-			
5. PTSD Symptoms	0.17^#^	0.37*	–0.49*	0.32*	-		
6. Pain Severity	0.21*	0.26*	–0.09	0.75*	0.31*	-	
7. Age	0.01	–0.06	0.17^#^	0.35*	–0.16^#^	0.25*	-

*p < 0.05; ^#^*p* ≤ 0.10.

## Discussion

The present study highlights how individuals in a comorbid chronic pain and PTSD group endorse more CV-19 Threat and Impact compared to healthy individuals, those with chronic pain only, and those with PTSD only. These findings are comparable to prior research by Benedict et al. (2020) wherein individuals with comorbid chronic pain and PTSD endorsed more depression, pain severity, and pain-related disability compared to individuals with chronic pain only. Our findings also show how authenticity may have protected individuals in the chronic pain only group from CV-19 Threat compared to those in the healthy group.

We hypothesized that authenticity would attenuate the CV-19 effects dependent on group. Regarding CV-19 Threat, the main effect of authenticity was non-significant, suggesting that authenticity was not a resilience factor overall for CV-19 Threat. However, results suggested that authenticity as a resilience factor of CV-19 Threat was unique to individuals with chronic pain in the absence of PTSD. The reason for null associations between authenticity and CV-19 Threat in the healthy group, PTSD only group, and comorbid group is unclear. CV-19 Threat represents anxiety and worry related to the CV-19 pandemic (Conway et al., 2020). Thus, the construct is cognitively and emotionally driven. Healthy individuals have many skills at their disposal un-disrupted by pain or PTSD (e.g., positivism and cognitive re-appraisal). Importantly, these unencumbered skills combined with lower allostatic load may make authentic living less of a factor in helping manage novel stressors for these individuals. Comparatively, individuals with chronic pain have potentially experienced *inauthenticity* for years due to the effects of their pain. As a result, individuals with chronic pain may be more likely to recognize authenticity as an important component of psychological health, monitor their feelings of authenticity (or lack thereof), and alter their cognitions, behaviors, and/or emotions when they do *not* feel authentic. While chronic pain may indeed impact how individuals perceive the world, PTSD is defined by alterations in mood and cognitions (American Psychiatric Association [APA], 2013). These alterations may prevent the use of effective self-affirming coping strategies (Vail et al., 2019, 2020) and thus, preventing individuals from harnessing the positive effects of living authentically. Moreover, authenticity is predicated on the *feeling* of being authentic and the ability to engage in behaviors that project those feelings (Wood et al., 2008; Lenton et al., 2013). The psychological need for feeling authentic is rooted in the idea that each individual has a primary “true self” that determines who they are, a concept known as psychological essentialism (Rivera et al., 2019). Due to the effects of pain, individuals with chronic pain may have been already altering their engagement with themselves and others prior to the pandemic, and managing what it means to be their “true self.” It is possible that aspects of PTSD, such as emotional numbing, avoidance of emotions, or distrust of self and others (Ehlers and Clark, 2000), prevent individuals from benefiting from feeling authentic and/or developing a new authentic “true self.” Indeed, authenticity is partially dependent on the *feeling* of authenticity (Wood et al., 2008; Lenton et al., 2013). If individuals with PTSD avoid certain stimuli, do not fully engage emotionally and cognitively with themselves or others, and struggle to trust themselves or others, it is more likely that they will not engage authentically and have a sense of their “true self.” The present work suggests that, although individuals with chronic pain may also struggle to recognize their emotions (Aaron et al., 2019), the addition of PTSD adds a layer that may prevent one’s authentic self from buffering CV-19 Threat.

Qualitative research and theoretical considerations highlight how individuals with chronic pain are forced to alter how they engage with the world, potentially causing identity-related distress (Smith and Osborn, 2007; Morley, 2008; Reed et al., 2021). Chronic pain compels individuals to find new ways of engaging authentically. If this is done effectively, then differences in authenticity between those with and without chronic pain may not be present. Also, this may account for the significant covariation between authenticity and CV-19 Threat in the chronic pain only group.

CV-19 Impact is conceptualized as being burdened financially or psychologically by the CV-19 pandemic (Conway et al., 2020). At first glance, authenticity did not appear to be associated with CV-19 Impact in any group. However, the standardized coefficients for all groups regarding the relationship between authenticity and CV-19 Impact ranged from -0.30 to -0.25, except for the PTSD only group, suggesting power may be an issue (data not presented). That this relationship was only.01 in the PTSD only group supports the idea that something unique to PTSD provides a barrier to using authenticity as a resilience factor. However, this does not account for the fact the comorbid group (which includes PTSD) had a standardized beta weight of -0.25.

Social distancing measures and protective face gear (e.g., masks) alter how to engage with others and society. For individuals with chronic pain, who are prone to higher levels of anxiety and depression (Gómez Penedo et al., 2020), there may be added anxieties around how to engage in a novel environment that the CV-19 pandemic imposes. However, it may also provide a means to re-evaluate current circumstances and engage in life in a new and more adaptive manner (Bland, 2020).

### Clinical Implications

The present research also suggests that focusing on authenticity among chronic pain patients may be clinically beneficial. Non-pharmacological behavioral treatments often target pain symptoms through the acceptance of pain, altering pain-specific thoughts, and creating narratives around their pain that are ego-syntonic (Williams et al., 2012; Carr et al., 2017; Hughes et al., 2017; Chow and Fok, 2020). A common thread in these treatments is the pursuit of self-transcendence and a search for meaning. The search for transcendence is the search for meaning in something outside of the self (e.g., volunteering and helping others, spirituality, and/or religion; Wong, 2020). Pandemics and other adverse events may limit these kinds of opportunities, making the search for transcendence more relevant (Wong, 2020). Congruent with this perspective, there has been a recent push to incorporate spiritual and existential themes into the treatment of chronic pain (Dezutter et al., 2016). Indeed, patients with chronic pain note that existential satisfaction is related to pain severity and disability (Dezutter et al., 2016). A recent clinical trial showed that pain-related disability improved when an existential component was added to cognitive-behavioral therapy, compared to cognitive-behavioral therapy alone (Gebler and Maercker, 2014).

Existential-humanistic perspectives incorporate both the joyful (e.g., accomplishments, love, and awe) and anxiety-provoking realities of being human (e.g., death, sickness, and suffering) into their conceptualization (Schneider, 2008; Schneider and Längle, 2012; Wong, 2021). For instance, the novel existential positive psychology perspective posits that well-being is the result of self-transcendence, and self-transcendence means simultaneously considering one’s suffering and accomplishments (Wong, 2021). Similarly, individuals with chronic pain often experience shame as a result of their physical pain (Smith and Osborn, 2007). From an existential positive psychology perspective, well-being (and “wholeness”) rely on acknowledging this physical pain and corresponding suffering while taking responsibility for finding new ways to engage meaningfully in life, connect with others, and maintain a spiritual and/or philosophical component of life (Wong, 2018, 2021). Therapy, then, can focus on finding ways to engage authentically while targeting these themes.

Other approaches offer different perspectives but remain faithful to not avoiding the realities of human suffering and physical pain. Research suggests acceptance and commitment therapy (ACT) and mindfulness approaches have shown to be effective at treating chronic pain outcomes (Veehof et al., 2011). ACT is a values-based approach that engages patients in determining what is important to them and then focuses on acting on these values (Hayes et al., 2012). In this way, ACT encourages individuals to engage authentically with the world and others. Mindfulness approaches are present-focused and teach individuals to “let go” of thoughts and emotions as being truly representative of themselves. In this way, mindfulness approaches de-emphasize rigid thought patterns and allow individuals to get more in tune with their authentic self.

### Limitations and Future Directions

The present study has limitations and future directions. As a cross-sectional design, causality cannot be established, and longitudinal designs attempting to parse out causal effects are indicated. With only 110 individuals completing Time 2, the null results may also be due to insufficient power (as suggested above). Although we intended to recruit individuals who were diagnosed with chronic pain and PTSD, standardized clinical interviews were not completed. Thus, it is possible that individuals would not qualify for these diagnoses through clinical interviews. The study sample lacked diversity regarding race, sexual orientation, and education. Previous research shows that people of color and lower social-economic status are more severely affected by the pandemic (Czeisler et al., 2020). The present study’s results should be interpreted in light of these generalizability restrictions. There is a plethora of evidence showing how the current medical systems in place disproportionately negatively affect certain marginalized populations (Liburd et al., 2020). The present work generally fails to include these populations, and more research is indicated to better understand the impact of the pandemic within these populations. Using a 21% pain disability cutoff score for the chronic pain only and comorbid group, combined with the average pain severity scores of 3.65 (chronic pain only) and 3.59 (comorbid group) point to a sample that is not as severe as other chronic pain populations. Similarly, the average PTSD scores for the PTSD only group were 30.09, and the average score for the comorbid chronic pain and PTSD only group was 38.23. These scores indicate the sample may not be as severe as other PTSD populations. Future research should include participants who are more severe, as results in these populations may vary from present results. Finally, correlations are presented with the caveat that the study design was such that specific groups of individuals were targeted for recruitment. Specifically, each participant group reflects a limited range of pain severity and PTSD symptoms. Therefore, interpreting correlations involving pain severity, pain-related disability, and PTSD symptoms in individuals who do not have chronic pain and/or PTSD is of limited utility.

## Conclusion

The present study examined authenticity as a resilience factor during the CV-19 pandemic. Participants were allocated to four separate groups based on their level of pain disability and PTSD symptom severity: healthy, chronic pain only, PTSD only, and comorbid chronic pain and PTSD. The comorbid group endorsed higher levels of CV-19 Threat and Impact compared to all other groups. There were no other between-group differences in CV-19 Threat and Impact. Importantly, greater authenticity was associated with less CV-19 Threat in the chronic pain only group, and not in any other group. As non-pharmacological chronic pain therapies focused on values, identity, acceptance, and internal narratives gain more popularity, incorporating elements designed to boost authenticity may play an important role. Results are interpreted within the limits of generalizability, as the present sample included a majority of White and educated participants.

## Data Availability Statement

The original contributions presented in the study are publicly available. This data can be found here: https://osf.io/zub97/.

## Ethics Statement

The studies involving human participants were reviewed and approved by the University of Texas Health Science Center at San Antonio. Written informed consent for participation was not required for this study in accordance with the national legislation and the institutional requirements.

## Author Contributions

DR contributed to research design, data collection, data analysis, and manuscript preparation. EL, BC, KV, PN, PH, and MG contributed to research design and manuscript preparation. DM contributed to data collection and research design. All authors contributed to the article and approved the submitted version.

## Conflict of Interest

The authors declare that the research was conducted in the absence of any commercial or financial relationships that could be construed as a potential conflict of interest.

## Footnotes

1 https://osf.io/hsyxv

2 https://osf.io/a35m4/

3 https://osf.io/9zbe2
